# A transformation uncertainty and multi-scale contrastive learning-based semi-supervised segmentation method for oral cavity-derived cancer

**DOI:** 10.3389/fonc.2025.1577198

**Published:** 2025-05-09

**Authors:** Ran Wang, Chengqi Lyu, Lvfeng Yu

**Affiliations:** Department of Stomatology, Shanghai Sixth People’s Hospital Affiliated to Shanghai Jiao Tong University School of Medicine, Shanghai, China

**Keywords:** pathological image segmentation, semi-supervised learning, oral cavity-derived cancer, contrastive learning, uncertainty estimation

## Abstract

**Objectives:**

Oral cavity-derived cancer pathological images (OPI) are crucial for diagnosing oral squamous cell carcinoma (OSCC), but existing deep learning methods for OPI segmentation rely heavily on large, accurately labeled datasets, which are labor- and resource-intensive to obtain. This paper presents a semi-supervised segmentation method for OPI to mitigate the limitations of scarce labeled data by leveraging both labeled and unlabeled data.

**Materials and methods:**

We use the Hematoxylin and Eosin (H&E)-stained oral cavity-derived cancer dataset (OCDC), which consists of 451 images with tumor regions annotated and verified by pathologists. Our method combines transformation uncertainty and multi-scale contrastive learning. The transformation uncertainty estimation evaluates the model’s confidence on data transformed via different methods, reducing discrepancies between the teacher and student models. Multi-scale contrastive learning enhances class similarity and separability while reducing teacher-student model similarity, encouraging diverse feature representations. Additionally, a boundary-aware enhanced U-Net is proposed to capture boundary information and improve segmentation accuracy.

**Results:**

Experimental results on the OCDC dataset demonstrate that our method outperforms both fully supervised and existing semi-supervised approaches, achieving superior segmentation performance.

**Conclusions:**

Our semi-supervised method, integrating transformation uncertainty, multi-scale contrastive learning, and a boundary-aware enhanced U-Net, effectively addresses data scarcity and improves segmentation accuracy. This approach reduces the dependency on large labeled datasets, promoting the application of AI in OSCC detection and improving the efficiency and accuracy of clinical diagnoses for OSCC.

## 1 Introduction

According to the World Cancer Research Fund’s International Report, over 377,700 cases of oral cavity-derived cancer were reported globally in 2020, ranking 16th among all cancers (1). Oral squamous cell carcinoma (OSCC) is a common and aggressive oral tumor, with a five-year survival rate of only around 50% (2). Pathological images are considered the gold standard for diagnosing and grading cancer (3), and their accurate interpretation is crucial for OSCC treatment and control. OSCC histopathological evaluation typically involves processes like formalin fixation, sectioning, paraffin embedding, and hematoxylin and eosin staining, followed by microscopic examination by trained pathologists (4). Pathologists use standardized criteria to assess the tumor’s presence, subtype, and other histological features.

Recent advancements in computer-aided systems, driven by high-precision imaging and computational power, have accelerated the development of automated methods for histopathological image analysis. Deep learning, in particular, has shown great promise in the automated segmentation of oral cavity-derived cancer images (OPI) (5–8). While these methods have shown promising results, they typically require large annotated datasets. However, pathological images, compared to other medical imaging modalities like MRI and CT, often have high spatial resolution, making accurate labeling more challenging. Additionally, the labeling process requires specialized knowledge and extensive diagnostic experience, making it difficult to obtain sufficient labeled data, which limits the broader application of deep learning methods for OPI segmentation.

Semi-supervised learning (SSL) addresses the challenge of limited labeled data by combining a small amount of labeled data with a large volume of unlabeled data. In medical image segmentation, consistency regularization methods are commonly used, assuming that small perturbations should not significantly change the model’s outputs. These methods introduce perturbations in data, model, and task, enforcing consistency across them. For data perturbation, techniques like Gaussian noise (9) and affine transformations (10) are often used. In model perturbation, methods such as Mean Teacher (MT) (11) have been effective, where Dropout operations in the teacher-student network and exponential moving averages (EMA) of model weights are used to improve model accuracy. To enhance prediction quality, uncertainty estimation techniques, such as prediction entropy (12), evidence theory (13), and KL divergence (14), have been incorporated. Multi-task consistency methods, such as reconstruction (15), boundary perception (16), and distance map tasks (17), are also used to better utilize unlabeled data.

The lack of large sample labels is the starting point for semi-supervised learning. In classical MT networks, the teacher model’s predictions are often used as pseudo-labels to guide the optimization of the student model. However, the substantial semantic gap between the pseudo-labels generated by the teacher model and the true labels can seriously impact the student model’s performance. Considering that pathologists typically rotate, flip, and otherwise transform pathological images in clinical practice to make a comprehensive evaluation, and that we desire deep networks to exhibit invariance (such as the ability to recognize objects under translation, rotation, scaling, or varying lighting conditions), we design a transformation-based uncertainty estimation (TB-UE) method. Building on UA-MT (12), we combine multiple data transformation methods to estimate uncertainty by measuring the model’s predictions for the same data point under different transformations. This method incorporates both data uncertainty and per-pixel entropy information, mitigating the detrimental effects of noisy pseudo-labels on the student model. However, this approach may lead to high similarity between the teacher and student models. To address this, we propose a multi-scale contrastive learning (MS-CL) method, which computes the average feature vectors of different categories from both the teacher and student models, using contrastive loss to pull together feature vectors of the same class and push apart those of different classes. This method not only alleviates the over-similarity problem between teacher and student models but also improves intra-class similarity and inter-class separability, resulting in more diverse feature representations. Additionally, we propose a boundary-aware enhanced U-Net (BAE-U-Net), which adds a boundary perception enhancement branch to the original U-Net (18), enabling the capture of boundary information in OSCC pathological images. In our BAE-U-Net, we design a channel-attention-based boundary-spatial feature fusion module (BSFM) that combines the boundary information extracted by the enhancement branch with the spatial information from U-Net, facilitating more comprehensive feature representation.

In summary, the contributions of this paper are as follows:

1. A semi-supervised segmentation method for Oral Cavity-derived Cancer pathological images is proposed, based on transformation uncertainty and multi-scale contrastive learning, and is designed to alleviate the limitations imposed by the scarcity of labeled data.
2. A transformation-based uncertainty estimation method is introduced, in which pixel uncertainty is estimated by evaluating the model’s predictions on data transformed using different methods.
3. A multi-scale contrastive learning method is presented, which improves intra-class similarity and inter-class separability while mitigating the over-similarity problem between the teacher and student models.
4. A boundary-aware enhanced U-Net is proposed, which integrates boundary information with spatial information to facilitate more comprehensive feature learning.
5. Extensive experiments on the dataset demonstrate the superiority of the proposed method compared to other approaches, highlighting its potential in addressing the issue of labeled data scarcity.

## 2 Related work

### 2.1 Semi-supervised segmentation of medical images

The main goal of semi-supervised learning is to utilize a large amount of unlabeled data to improve supervised learning performance. In medical image segmentation, consistency regularization and pseudo-labeling are two major paradigms for semi-supervised learning.

Pseudo-labeling methods typically involve training a model on a labeled dataset, then using this trained model to assign pseudo-labels with confidence scores to unlabeled data. High-confidence pseudo-labels are added to the labeled set to enhance model performance. Self-training and co-training are two common approaches in pseudo-labeling. Self-training focuses on refining pseudo-labels using various strategies to make them closer to true labels. For instance, Bai et al. (19) optimized pseudo-labels with conditional random fields, while Zeng et al. (20) selected high-confidence samples by combining class information and prediction entropy. However, single-branch self-training methods can be unstable due to variations in pseudo-label quality. Co-training, derived from multi-view learning, uses multiple complementary views of the data for multi-branch training. High-confidence predictions are added to other branches’ data or consistency methods are applied to guide interaction between branches. Examples of multi-view approaches include adversarial learning to generate multiple views (21), multi-modality data for multi-view samples (22), and multi-branch Transformer-CNN structures for feature extraction (23). In particular, CNN-based branches are widely used due to their powerful local feature extraction capability, which complements global dependencies captured by Transformer modules, and improves the model’s robustness to variations in tissue structures and staining.

Consistency regularization methods are based on the smoothness assumption, adding perturbations to data points in terms of data, model, and task, and enforcing consistency. Significant progress has been made in data perturbation consistency, with methods like patch-shuffling (24), cut-paste augmentation (25), and Copy-Paste (26). For model perturbation consistency, besides the Mean Teacher (MT) network, multi-decoder structures (27–29) are also effective. These structures use a shared encoder and multiple decoders, which either learn from each other or minimize statistical differences between decoders to reduce model uncertainty. Other model perturbation methods include multi-scale consistency (30), complementary consistency (31), and the use of anatomical prior knowledge (32).

### 2.2 Pathological image segmentation

The goal of pathological image segmentation is to divide the image into different components, such as cell nuclei, glands, or tissue regions, which is essential for clinical diagnosis. By linking morphological features to clinical outcomes, segmentation provides an objective and quantitative analysis that helps guide treatment decisions. Deep learning-based methods have shown great promise in the segmentation of pathological images, but they typically rely on pixel-level labels, which are time-consuming and expensive to obtain.

In breast cancer pathological image analysis, Li et al. (33) proposed DeepTree, a deep learning architecture based on a tree-structured diagnostic strategy. This method represents relationships between different pathological categories and establishes a new framework for segmentation in pathological regions of interest (ROI). In lung cancer diagnosis, Chen et al. (34) introduced a weakly supervised learning method using a deep generative model to convert fluorescent tissue images into virtual H&E stained images, followed by a multi-instance learning model for segmentation. This approach leverages weak supervision to mitigate the need for large labeled datasets. For bladder cancer analysis, He et al. (35) developed MultiTrans, a framework that enhances segmentation accuracy through multi-scale feature fusion, aiding in the segmentation of head-and-neck at-risk organs.

These studies showcase the application of CNNs, graph convolutions, and Transformers in pathological image segmentation, as well as the growing use of weakly supervised and unsupervised methods to reduce the reliance on large annotated datasets.

## 3 Methods

### 3.1 Overview

The OPI semi-supervised segmentation task aims to jointly train a model using a large amount of unlabeled data and a small amount of labeled data to improve model performance. We use a dataset D consisting of M labeled samples and NN unlabeled samples, where M≪N . The labeled dataset is defined as $D^{L}={\left\{X^{i},Y^{i}\right\}}_{i=1}^{M}$ , and the unlabeled dataset is defined as $D^{U}={\left\{X^{i}\right\}}_{i=M+1}^{M+N}$ , where $X^{i}\in {\mathbb{R}}^{H\times W\times C}$ represents a pathological image of height H, width W, and C channels, and $Y^{i}\in {\mathbb{R}}^{H\times W}$ represents the corresponding label map for $X^{\text{i}}$ . The goal of the semi-supervised segmentation task is to learn a student model $f_{s}\left(\theta_{s}\right)$ parameterized by $\theta_{s}$ from the dataset D, such that each pixel in the input image is mapped to its correct class.

**Figure 1:**
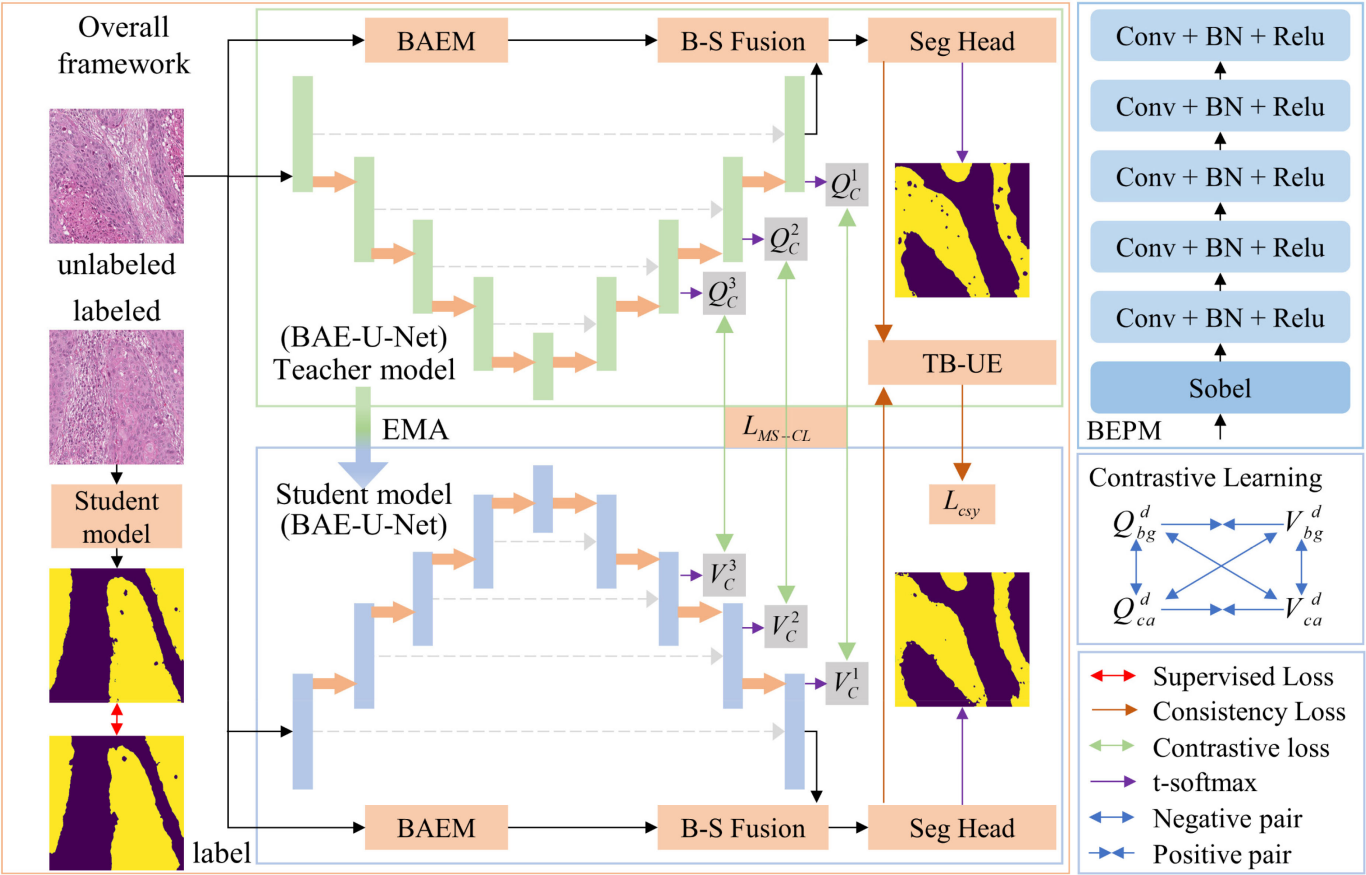
Overall framework of the proposed method for OPI semi-supervised segmentation based on transformation uncertainty and multi-scale contrastive learning.


**Figure 1** illustrates the OPI semi-supervised segmentation method based on transformation uncertainty and multi-scale contrastive learning, which is proposed in this paper. This method aims to jointly train on a small amount of labeled data and a large amount of unlabeled data to mitigate the limitation caused by the shortage of labeled data in OPI segmentation models. The method follows the approach of MT (10). Specifically, the network is divided into a teacher model and a student model, both of which share the same network architecture. The parameters $\theta_{s}$ of the student network are updated using the gradient backpropagation algorithm. For the teacher model, the parameters $\theta_{T}$ are updated using the EMA method, formulated as:

(1) $$\theta_{T}^{t}=\alpha \theta_{T}^{t-1}+\left(1-\alpha \right)\theta_{s}^{t}$$

where $\theta_{T}^{t}$ denotes the parameters of the teacher model after the t-th iteration, $\theta_{s}^{t}$ denotes the parameters of the student model after the t-th iteration, and α is the EMA decay coefficient controlling the rate at which the teacher model parameters are updated. To better utilize the multi-scale information of pathological images, we design a contrastive learning method by optimizing the multi-scale contrastive loss function $L_{MS-CL}$ to distinguish the multi-scale class features of OPI. Additionally, we incorporate an uncertainty estimation method that combines data transformation and entropy, building on MT, to reduce the gap between teacher model predictions and true labels. Based on this, the consistency loss $L_{csy}$ is optimized to enhance the prediction accuracy of the student model. Below, we provide a detailed explanation of the proposed BAE-U-Net, the TB-UE method, and the MS-CL method.

### 3.2 BAE-U-Net

To extract boundary information for OPI and combine it with the spatial information extracted by the U-Net, we propose the BAE-U-Net. This network consists of the classical U-Net, a boundary-enhancement branch, and a BSFM. In this paper, the final convolutional layer of the U-Net is referred to as the “seg-head,” while the remaining parts are referred to as the “seg-net.” The input data pass through the seg-net to obtain the spatial features $F_{S}\in {\mathbb{R}}^{H\times W\times C_{S}}$ . The structure of the boundary-aware enhancement branch is shown in **Figure 1**. After passing through this branch, the enhanced boundary features $F_{BE}\in {\mathbb{R}}^{H\times W\times C_{B}}$ are obtained. This structure consists of boundary-aware and boundary-enhancement components, aiming to capture a more comprehensive boundary feature representation of the pathological image.

**Figure 2:**
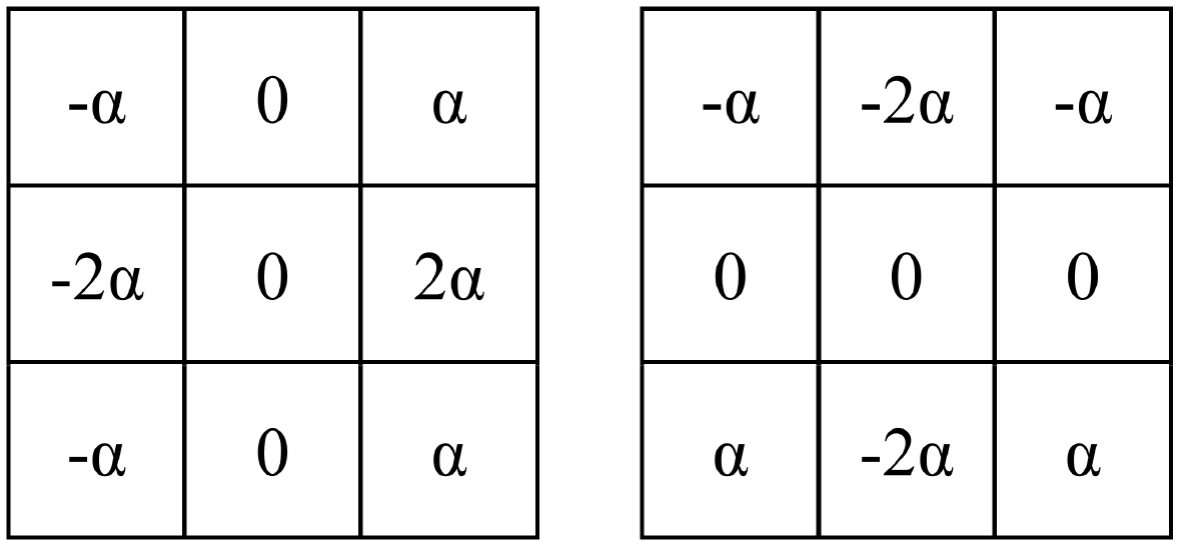
Sobel operator used in the boundary-aware module.

1. Boundary-Aware Module: This module consists of two Sobel operators, as illustrated in **Figure 2**. These operators are used to extract the horizontal and vertical boundary information of the image. The parameter α is a learnable parameter.
2. Boundary-Enhancement Module: This module consists of five feature extraction layers, each of which is formed by sequentially connecting a convolutional layer, a batch normalization layer, and a ReLU activation layer. The kernel sizes of the convolutional layers are 5, 5, 3, 3, and 1. This module retains a significant amount of boundary detail and further reduces the impact of noise and artifacts in the image, achieving the goal of refining and enhancing the image boundary features.

**Figure 3:**
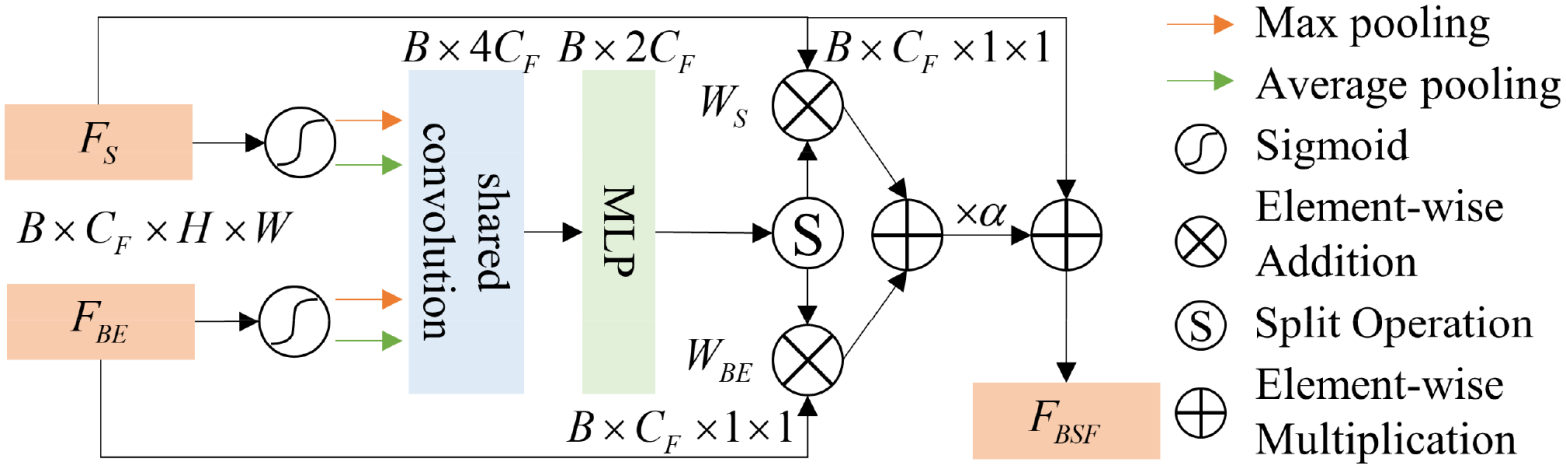
BSFM.


To better fuse boundary features with spatial features and avoid information loss caused by traditional addition or multiplication methods, we propose a BSFM, the structure of which is shown in **Figure 3**. Considering the semantic gap between boundary information and global spatial information, we implement cross-modal fusion of spatial and boundary information in the form of channel attention. The fused features can be formulated as (Equation 2):

(2) $$F_{BSF}=F_{S}⊕\left(\alpha \times \left(\left(F_{S}⊗W_{S}\right)+\left(F_{BE}⊗W_{BE}\right)\right)\right)$$

where $F_{BSF}$ denotes the boundary-spatial joint features, and α is a parameter that adjusts the importance of boundary information and spatial information. $W_{S}$ and $W_{BE}$ represent the weights of spatial and boundary information, which are obtained through the cross-modal attention mechanism. As shown in **Figure 3**, the cross-modal attention mechanism structure includes an activation function ( σ ), max-pooling (MP), average-pooling (AP), shared convolution (CC), and multi-layer perceptron (MLP). The process of obtaining the boundary-spatial joint weights $W_{BES}$ can be represented as (Equation 3):


(3) $$W_{BES}\text{=MLP}\left(Concat\left(\begin{array}{l} CC\left(AP\left(\sigma \left(F_{S}\right)\right)\right),CC\left(MP\left(\sigma \left(F_{S}\right)\right)\right),\\ CC\left(AP\left(\sigma \left(F_{BE}\right)\right)\right),CC\left(MP\left(\sigma \left(F_{BE}\right)\right)\right) \end{array}\right)\right)$$

For both boundary features and spatial features, we first apply the sigmoid activation function, followed by MP and AP for channel attention, and use shared convolutions for processing. For the convolutional features, we initially fuse the two feature types using the concatenation operation (Concat). To capture the complex relationships between channels and the interactions of features, we apply MLP to the fused features, resulting in the boundary-spatial joint weights. Since $C_{S}=C_{B}$ in this paper, the effectively compressed and fused feature weights are split into equal-sized $W_{S}$ and $W_{BE}$ to distinguish the importance of spatial and boundary information.

### 3.3 TB-UE

To further mitigate the issue of incorrect predictions in the student model due to noisy labels, we propose an uncertainty estimation method based on data transformations, building upon the UA-MT method. This method effectively combines pixel entropy information with data transformation invariance, specifically divided into pixel entropy estimation and transformation invariance estimation.

**Figure 4:**
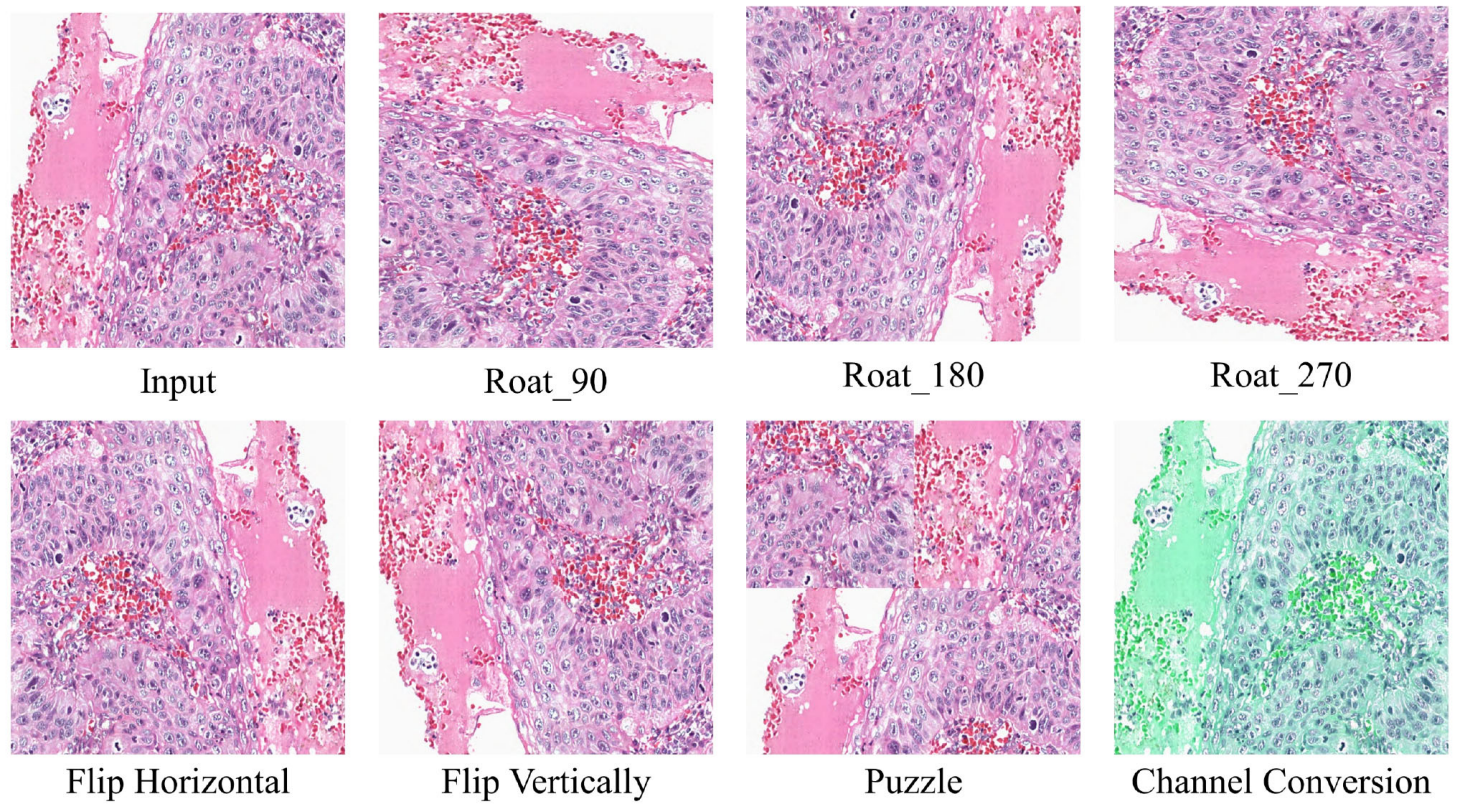
Illustration of different types of data transformations applied to the original data.


1. Pixel Entropy Estimation: We adopt the method from UA-MT to calculate the entropy of each pixel in the pseudo-labels. First, we perform T forward passes of the data through the teacher model to simulate Monte Carlo sampling. Let $p_{t}^{c}$ represent the predicted probability for class c at pixel i during the t-th forward pass. Then, the sum of probabilities over all classes is: $\sum_{c=1}^{n}p_{t}^{c}=1$ , where n is the total number of classes. The average predicted probability for class cc across the T forward passes is: $u_{c}=\left(\sum_{t=1}^{T}p_{t}^{c}\right)/T$ The entropy at pixel ii is then calculated as: $Q_{e}\left(i\right)=-\sum_{c=1}^{n}u_{c}logu_{c}$ . Next, we filter the high-confidence pixels based on the entropy values. We define a Boolean function ${\left\{condition\right\}}_{1}$ , which outputs 1 when the condition is true, otherwise 0. The high-confidence entropy mask $Mask_{E}$ is defined as: $Mask_{E}={\left\{Q_{e}\left(i\right)<\xi_{E}\right\}}_{1}$ , where $\xi_{E}$ is the uncertainty threshold for pixel entropy, which varies over iterations.
2. Transformation Uncertainty Estimation: To better estimate transformation uncertainty, we design M=7 data transformation methods, which include rotation (90°, 180°, and 270°), flipping (horizontal and vertical), patching (36), and color channel transformations. **Figure 4** illustrates the results of the different transformation methods. The transformed data can be represented as: $T_{j}=Trans_{j}\left(X\right),j\in \left[1,7\right]$ , where $Trans_{j}$ denotes the j-th data transformation method. After applying these transformations to the data, we pass them through the teacher model and reverse the transformations to obtain the transformed predictions, represented as: $Y_{j}^{T}=ITrans_{j}\left(f_{T}\left(T_{j}\right)\right)$ , where $ITrans_{j}$ represents the inverse transformation corresponding to $Trans_{j}$ , and $Y_{j}^{T}$ denotes the predicted labels after the inverse transformation. We then compute the transformation confidence map for each pixel, defined as: $Q_{T}^{c}\left(i\right)=M_{C}/M$ , where $M_{C}$ represents the number of times that pixel i is predicted as class cc across the transformations. The high-confidence transformation mask $Mask_{T}$ is defined as (Equation 4):

(4) $$Mask_{T}={\left\{\left(Q_{T}^{1}\left(i\right)\;\vee \;Q_{T}^{T}\left(i\right)\right)\cdot \cdot \cdot \vee Q_{T}^{T}\left(i\right)>\xi_{T}\right\}}_{1}$$

where ∨ denotes the pixel-wise OR operation, and $\xi_{T}$ is the uncertainty threshold for transformation invariance.

We calculate the consistency loss $L_{csy}$ between the teacher and student models using the obtained masks $Mask_{E}$ and $Mask_{T}$ (Equation 5):


(5) $$L_{csy}=\frac{{\sum_{i}Mask\left(i\right)\left\|P_{seg}^{s}-P_{seg}^{T}\right\|}^{2}}{\sum_{i}Mask\left(i\right)}$$

where $P_{seg}^{s}$ and $P_{seg}^{T}$ represent the predictions from the student and teacher models, respectively. The mask Mask is the pixel-wise product of $Mask_{E}$ and $Mask_{T}$ , which integrates both pixel entropy and transformation invariance information. This combined mask effectively constrains the pseudo-labels, alleviating the performance degradation caused by semantic gaps between pseudo-labels and true labels.

### 3.4 MS-CL

The consistency loss method proposed in Section 3.3 works well for constraining the similarity between the predictions of the student and teacher models, but it lacks a mechanism to enforce intra-class compactness and inter-class separability, which can lead to over-mixing of the features between the student and teacher models. Therefore, in this section, we propose a MS-CL method to enhance the intra-class similarity and inter-class separability of features, while also de-mixing the student and teacher models.

The flow of the MS-CL method is shown in **Figure 1**. We calculate the multi-scale class contrastive learning loss $L_{MS-CL}$ based on the outputs from the last D=3 layers of the seg-net. For the output features at scale d, $F_{S}^{d}\in {\mathbb{R}}^{C_{d}\times H_{d}\times W_{d}}$ , we first apply a convolution operation with a kernel size of 1 to transform them into features $F_{S}^{d^{\prime }}\in {\mathbb{R}}^{C_{out}\times H_{d}\times W_{d}}$ is the output channel size. For the teacher model, we compute the average feature vector for each class (Equation 6):


(6) $$Q_{c}^{d}=\frac{\sum_{n=1}^{H_{\text{d}}\times W_{d}}F_{S}^{d^{\prime }}{\tilde{P}}_{c,n}^{d}}{\sum_{n=1}^{H_{\text{d}}\times W_{d}}{\tilde{P}}_{c,n}^{d}}$$

where $Q_{c}^{d}$ represents the average feature vector for class cc at scale d, and ${\tilde{P}}_{c,n}^{d}$ is the probability that pixel n at scale dd belongs to class c, which is obtained by applying the softmax function to $F_{S}^{d}\in {\mathbb{R}}^{C_{d}\times H_{d}\times W_{d}}$ . Similarly, for the student model, we compute the average feature vector $V_{c}^{d}$ . Since our dataset contains only background and target (oral tumor region) classes, the average feature vectors at scale dd for both the teacher and student models are denoted as $Q_{bg}^{d}$ , $Q_{ca}^{d}$ , $V_{bg}^{d}$ and $V_{ca}^{d}$ . As shown in **Figure 1**, to calculate the multi-scale contrastive loss, we first compute the contrastive loss $L_{CL}^{d}$ at each scale.

Since our goal is to bring the feature vectors of the same class closer and push the feature vectors of different classes apart, we use the InfoNCE loss function. The contrastive loss at scale d isdefined in Equation 10. where τ is the temperature parameter, and sim(a,b) represents the similarity between vectors a and b. The multi-scale contrastive loss is defined as (Equation 7):


(7) $$L_{MS-CL}=\sum_{d=1}^{D}\beta^{d}\cdot L_{CL}^{d}$$

### 3.5 In this paper, $\beta^{d}$ represents the weight of the loss function at different scales, with the constraint: $\sum_{d=1}^{D}\beta^{d}=1$ In our method, we set $\beta^{1}=0.6,\beta^{2}=0.3,\beta^{3}=0.1$ Loss function

The loss function in this paper is composed of two parts: the supervised loss $L_{S}$ and the unsupervised loss $L_{U}$ . The supervised loss is calculated by averaging the cross-entropy loss and Dice loss between the student model’s predictions and the true labels over the labeled dataset $D_{L}$ . The unsupervised loss consists of the consistency loss $L_{csy}$ and the multi-scale contrastive loss $L_{MS-CL}$ . The unsupervised loss $L_{U}$ can be expressed as (Equation 8):


(8) $$L_{U}=\omega \cdot \left(L_{csy}+L_{MS-CL}\right)$$

where ω is a Gaussian weighting function defined as: $\omega =0.001\cdot \exp(-5\cdot {\left(1-t/t_{\max}\right)}^{2})$ . Here, t denotes the current iteration, and $t_{\max}$ represents the maximum number of iterations. Finally, the total loss function for our method can be written as (Equation 9):


(9) $$L=L_{s}+\lambda L_{u}$$

where λ is a pre-defined weight that balances the supervised loss and the consistency loss.


(10) $$\begin{array}{c} L_{CL}^{e}=-\log\\ \frac{\exp(\text{sim}(Q_{bg}^{d},V_{bg}^{d})/\tau )+\exp(\text{sim}(Q_{ca}^{d},V_{ca}^{d})/\tau )}{\exp(\text{sim}(Q_{bg}^{d},V_{bg}^{d})/\tau )+\exp(\text{sim}(Q_{bg}^{d},Q_{ca}^{d})/\tau )+\exp(\text{sim}(Q_{bg}^{d},V_{ca}^{d})/\tau )+\exp(\text{sim}(V_{bg}^{d},Q_{ca}^{d})/\tau )+\exp(\text{sim}(V_{bg}^{d},V_{ca}^{d})/\tau )} \end{array}$$

## 4 Experiments

### 4.1 Dataset

In this study, we use the Hematoxylin and Eosin (H&E) stained oral cavity-derived cancer dataset (OCDC) collected in (5). The tumor regions in this dataset have been manually annotated by experts and verified by pathologists. The OCDC dataset consists of 1,020 histological images with a size of 640×640 pixels, which include fully annotated tumor regions for segmentation purposes. All histological images were digitized at a 20× magnification. Since our experiment focuses on segmenting tumor regions, we excluded 569 images that contained no tumor areas, as confirmed by pathologists’ gold-standard annotations. The remaining 451 images were used for the experiments.

### 4.2 Evaluation metrics

To ensure a fair comparison of the proposed method with other methods, we used five common evaluation metrics to assess the performance of the proposed model and other approaches on the same test set: Overall Accuracy (OA), Average Accuracy (AA), Dice Similarity Coefficient (DSC), and Jaccard Index. The results for each method were summarized, and the average and standard deviation for each metric were reported in the table. OA measures the proportion of correctly predicted samples out of the total samples. AA is the average accuracy across all classes, emphasizing class balance. DSC and Jaccard evaluate the similarity between the segmentation results and the ground truth. The formulas for the four metrics are as follows (Equations 11–14):


(11) $$OA=\frac{TP+TN}{TP+TN+FP+FN}$$

(12) $$AA=\frac{1}{2}\left(\frac{TP}{TP+FN}+\frac{TN}{FP+TN}\right)$$

(13) $$DSC=\frac{2\times |A\cap B|}{\left|A|+|B\right|}$$

(14) $$Jaccard=\frac{\left|A\cap B\right|}{\left|A\cup B\right|}$$

where TP represents the number of samples correctly predicted as positive, TN represents the number of samples correctly predicted as negative, FP refers to the number of negative samples incorrectly predicted as positive (false positives), and FN refers to the number of positive samples incorrectly predicted as negative (false negatives). A is the foreground pixel set in the ground truth, and B is the foreground pixel set in the predicted result. |A∩B| represents the number of pixels in the intersection of the ground truth and predicted results, while |A∪B| represents the number of pixels in their union. |A| and |B| denote the number of foreground pixels in the ground truth and prediction, respectively. In addition, we also used 95% Hausdorff distance (HD95) to quantitatively evaluate the segmentation of the boundaries.

### 4.3 Implementation details

In this study, the proposed network was implemented using the PyTorch framework and trained on an NVIDIA GeForce RTX 3090 GPU. We used the SGD optimizer with a learning rate of 0.01 and a momentum coefficient of 0.9. 10% of the data was used as the test set, and 5-fold cross-validation was performed on the remaining 90%. The model was trained for 100 epochs. The model with the highest DSC on the validation set was selected as the final model for testing. During training, the batch size for both labeled and unlabeled data was set to 1, with equal proportions of labeled and unlabeled data. Based on the findings in MT (10), we set the EMA coefficient αin Equation 1 to 0.99.

### 4.4 Comparison experiments

#### 4.4.1 Comparison with fully supervised methods

**Figure 5:**
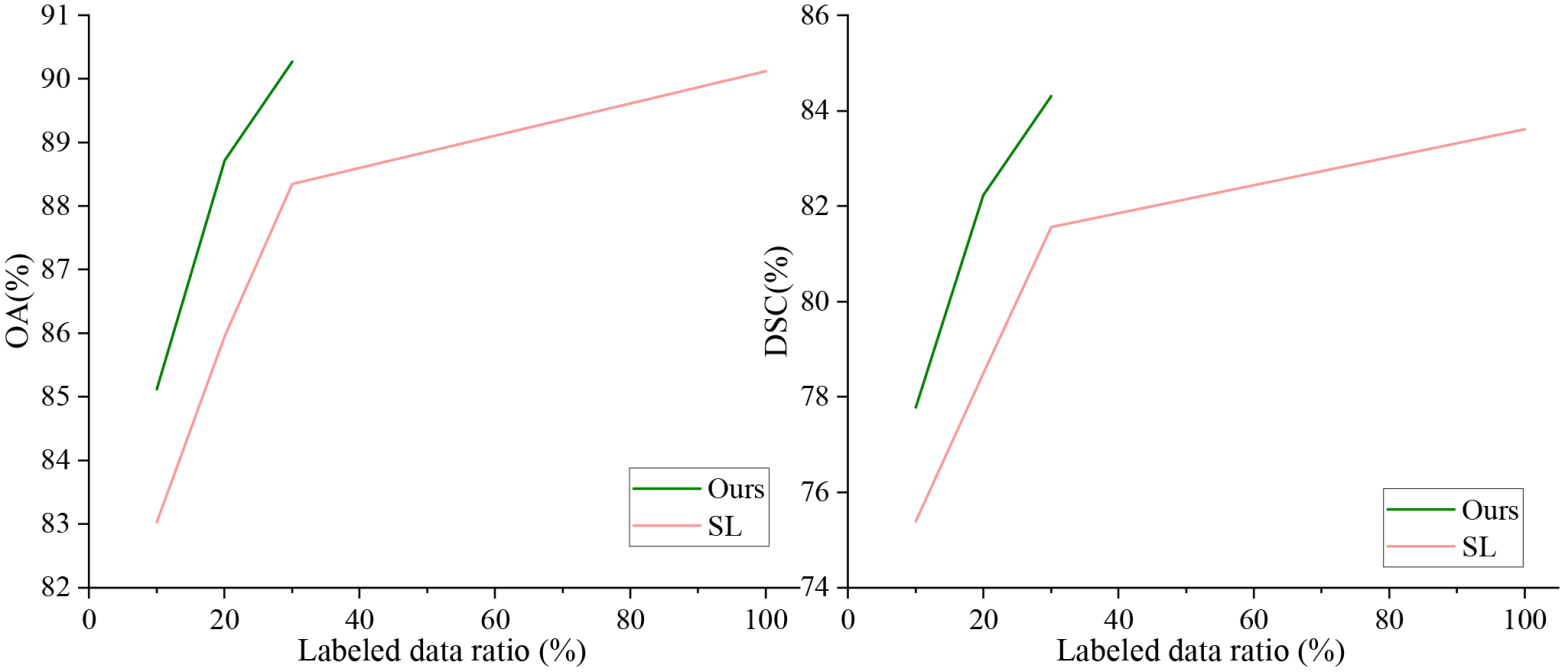
Visualization of OA and DSC using different proportions of labeled data on the OCDC dataset.

**Figure 6:**
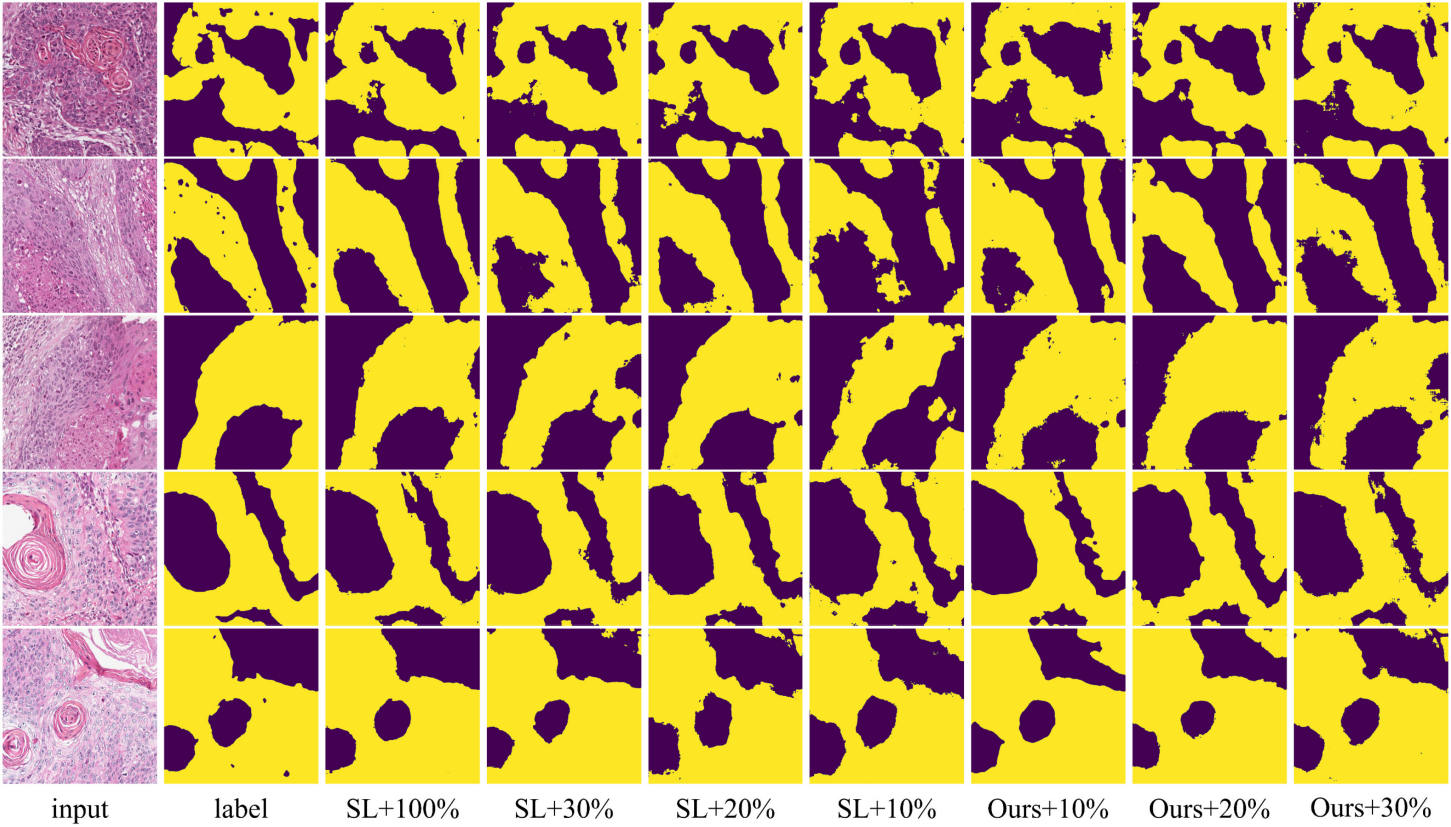
Visualization comparison of supervised and proposed methods using various proportions of labeled data on the OCDC dataset. Yellow regions represent tumor areas, and the rest are background.

To verify that our method can leverage unlabeled data to improve model segmentation performance, we trained the model using 10%, 20%, and 30% labeled data along with the corresponding proportion of unlabeled data, and compared the results with fully supervised methods. The quantitative experimental results are reported in **Table 1**. The data in **Table 1** shows that the proposed method outperforms the fully supervised methods in all five average metrics when using the corresponding proportions of labeled data. Specifically, the DSC improved by 2.39%, 3.74%, and 2.75%, respectively. Notably, when using 30% labeled data, the proposed method performed better than using 100% labeled data, indicating that the method significantly reduces the need for labeled data. **Figure 5** shows the visualization of OA and DSC. From the figure, it is clear that as the proportion of labeled data increases from 10% to 30%, the evaluation metrics show significant improvement in the fully supervised method. However, from 30% to 100%, the improvement in the metrics is not as pronounced, highlighting that simply increasing the amount of labeled data does not significantly improve model performance, further emphasizing the importance of semi-supervised learning on the OCDC dataset. To visually demonstrate the improvement of our method over the fully supervised approach, we show the visual output results in **Figure 6**. It can be seen that when only 10% labeled data is used, the fully supervised method fails to recognize a significant portion of the tumor region, while our method successfully identifies more tumor areas. Furthermore, the segmentation results from our method more accurately capture the tumor boundaries compared to the fully supervised method, demonstrating theaccuracy of the proposed boundary-aware enhancement module. This also explains why our method consistently outperforms the corresponding fully supervised methods in terms of HD95.

**Table 1:** Quantitative results of supervised and proposed methods using various proportions of labeled data on the OCDC dataset.

Method	Samples used		Metrics				
	M	N	OA(%) ↑	AA(%) ↑	DSC(%) ↑	Jaccard(%)↑	HD_95_ ↓
SL	324	0	90.12	84.59	83.61	73.94	11.48
	96	0	88.35	84.06	81.56	71.00	12.55
	65	0	85.96	81.56	78.49	67.78	13.60
	32	0	83.03	78.99	75.39	63.79	14.69
Ours	96	228	90.27	88.84	84.31	74.52	11.82
	65	259	88.72	88.26	82.23	75.82	12.10
	32	292	85.12	86.61	77.78	66.27	13.93

The results report the average from five-fold cross-validation experiments.

#### 4.4.2 Comparison with other semi-supervised methods

To prove the effectiveness of the proposed method in semi-supervised scenarios, we conducted comparison experiments on the GIN and CCA datasets using 10% and 20% labeled data. We compared our method with six state-of-the-art semi-supervised methods: MT (10), UA-MT (11), CCT (37), CPS (38), DMMT (31), and SPCL (39). The quantitative experimental results are reported in **Table 2**.

**Table 2:** Quantitative results of different methods using various proportions of labeled data on the OCDC dataset.

Method	Samples used		Metrics				
	M	N	OA(%) ↑	AA(%) ↑	DSC(%) ↑	Jaccard(%)↑	HD_95_ ↓
SL	324	0	90.12	84.59	83.61	73.94	11.48
	65	0	85.96	81.56	78.49	67.78	13.60
	32	0	83.03	78.99	75.39	63.79	14.69
MT	32	292	82.67	78.66	74.04	62.07	14.36
UA-MT	32	292	82.00	77.88	73.56	61.39	14.49
CPS	32	292	82.57	78.97	74.80	62.90	14.16
CCT	32	292	80.90	76.72	72.77	60.12	14.50
DMMT	32	292	81.43	77.92	72.51	60.00	14.03
SPCL	32	292	83.09	78.81	75.66	63.76	14.55
Ours	32	292	**85.12**	**86.61**	**77.78**	**66.27**	13.93
MT	65	259	87.03	82.66	80.14	69.15	13.18
UA-MT	65	259	85.51	80.89	77.43	66.38	13.58
CPS	65	259	87.10	82.76	79.69	68.87	13.26
CCT	65	259	83.82	80.12	76.44	65.25	12.87
DMMT	65	259	86.41	82.55	79.75	68.62	13.08
SPCL	65	259	86.15	80.64	76.88	66.06	13.30
Ours	65	259	**88.72**	**88.26**	**82.23**	**75.82**	12.10

The results report the average from five-fold cross-validation experiments, with the optimal results bolded and the suboptimal results underlined.

**Figure 7:**
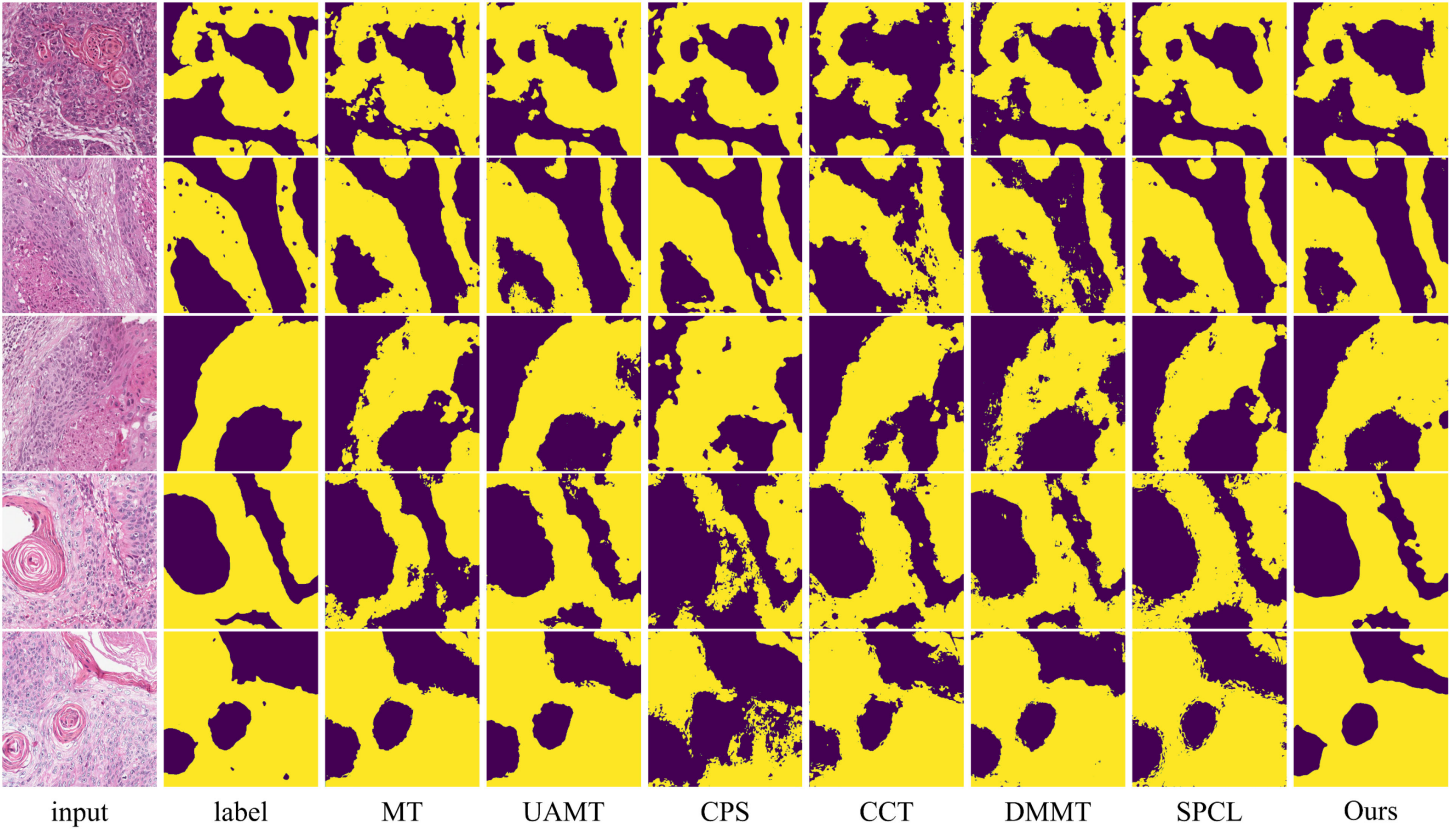
Visualization comparison of the proposed method with other semi-supervised methods using 10% labeled data on the OCDC dataset. Yellow regions represent tumor areas, and the rest are background.

**Figure 8:**
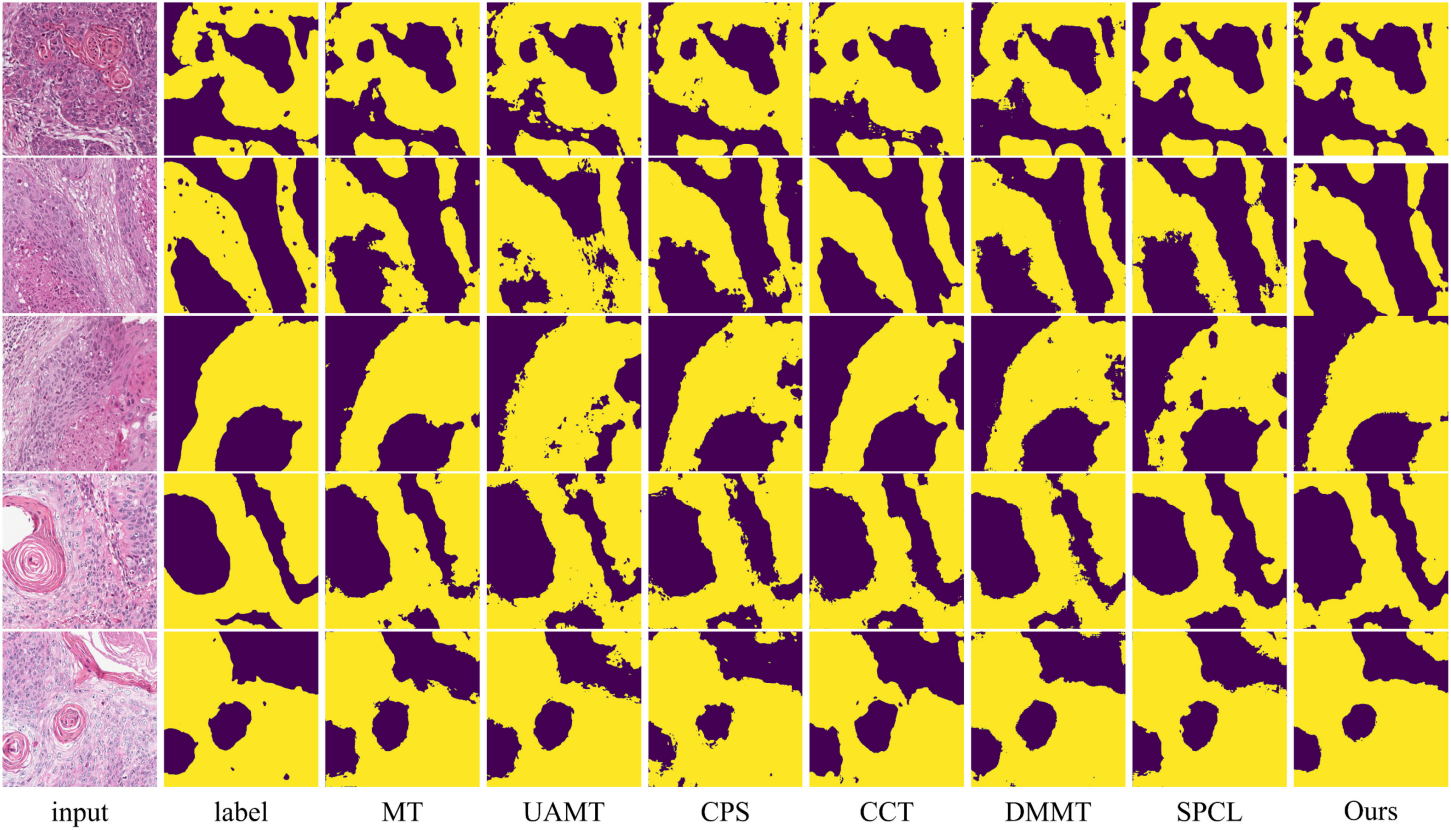
Visualization comparison of the proposed method with other semi-supervised methods using 20% labeled data on the OCDC dataset. Yellow regions represent tumor areas, and the rest are background.

The table shows that when using 10% labeled data for training, the six semi-supervised methods do not achieve significant improvements compared to the fully supervised method, especially the teacher-student network-based models (MT, UA-MT, etc.), whose segmentation results are worse than the fully supervised approach. Our method outperforms the second-best method across all five evaluation metrics by 2.03%, 7.64%, 2.12%, 3.37% and 0.2, respectively. In this setup, the SPCL method, which utilizes contrastive learning, achieves relatively good results among the other semi-supervised methods. However, when the labeled data increases to 20%, this advantage does not persist. In contrast, our method achieves the best results in both settings. **Figures 7**, **8** show the visual segmentation results for 10% and 20% labeled data. From the figures, it is evident that our method’s segmentation results are much closer to the ground truth, especially in terms of accurately delineating boundaries. Compared to the UA-MT method, which only uses entropy for uncertainty estimation, our method significantly reduces false positive samples, demonstrating that the proposed uncertainty estimation based on data transformation effectively reduces the teacher model’s prediction errors.

To further validate the effectiveness of the proposed method under limited annotation conditions, we conducted paired t-tests between our method and several baseline approaches (including fully supervised and representative semi-supervised methods) under two training scenarios using 10% and 20% labeled data. The significance testing results are summarized in **Table 3**. As observed, all p-values are less than 0.05, indicating that the performance improvements of our method over the baselines are statistically significant. These results demonstrate the robustness and superiority of the proposed method in low-label regimes.

**Table 3:** P-values of paired t-tests between the proposed method and other methods.

Samples used	Metrics	SL	MT	UA-MT	CPS	CCT	DMMT	SPCL
10%	OA	0.013	0.027	0.025	0.019	0.023	0.036	0.042
	DSC	0.020	0.031	0.029	0.022	0.022	0.041	0.038
20%	OA	0.010	0.029	0.037	0.039	0.038	0.046	0.029
	DSC	0.018	0.035	0.044	0.040	0.032	0.042	0.033

In addition, we evaluated the model complexity and inference time of all compared methods, and the results are reported in **Table 4**. Since the SL, MT, UA-MT, CPS, and SPCL methods all use a standard 2D U-Net as their feature extractor, they have similar numbers of parameters and inference times. In contrast, our method introduces the BAEM module, which slightly increases the model size and inference time.

**Table 4:** Parameter count and inference time on the entire dataset for the proposed method and comparative methods.

	Ours	SL	MT	UA-MT	CPS	CCT	DMMT	SPCL
Params(M)	1.83	1.81	1.81	1.81	1.81	1.82	1.71	1.81
Times(s)	92.23	67.85	69.02	67.94	68.43	73.21	73.32	69.21

### 4.5 Ablation study

In this section, we design experiments to validate the proposed methods, including the TB-UE approach, the MS-CL method, and the MAEM. The quantitative results of the experiments are reported in **Table 5**. Firstly, we constructed a baseline model, named “Basic”, which is based on the MT network, by removing the aforementioned three methods. As the uncertainty estimation methods, we removed consists of two parts: entropy- based uncertainty estimation (EU) and data transformation-baseduncertainty estimation (TU), we respectively added EU and TU to the Basic model. Notably, when the EU module is added, the network turns into the UA-MT model. The results in **Table 5** show a noticeable reduction in evaluation metrics when the EU module is added compared to the MT model. This suggests that relying solely on pixel entropy for uncertainty estimation is insufficient for the complex scenarios encountered in oral pathology image (OPI) segmentation. However, when the TU module is added on its own or in combination with the EU module, the evaluation metrics show significant improvement. This demonstrates that the TU module enhances the accuracy of pseudo-labels generated by the teacher model, and it confirms the complementarity between TU and EU modules.

**Table 5:** Quantitative results of ablation experiments using 20% labeled data on the OCDC dataset.

	Basic	EU	TU	BAEM	MS-CL	OA(%)↑	AA(%)↑	DSC(%)↑	Jaccard(%)↑	HD_95_ ↓
Model1	*√*					87.03	82.66	80.14	69.15	13.18
Model2	*√*	*√*				85.51	80.89	77.43	66.38	13.58
Model3	*√*		*√*			87.16	82.59	80.70	70.17	13.05
Model4	*√*	*√*	*√*			87.84	84.27	81.55	70.94	12.98
Model5	*√*	*√*	*√*	*√*		88.05	83.48	82.32	71.53	12.33
Model6	*√*	*√*	*√*		*√*	88.62	83.62	82.13	71.66	12.71
Model7	*√*	*√*	*√*	*√*	*√*	88.72	88.26	82.23	75.82	12.10

The results report the average from five-fold cross-validation experiments.

**Figure 9:**
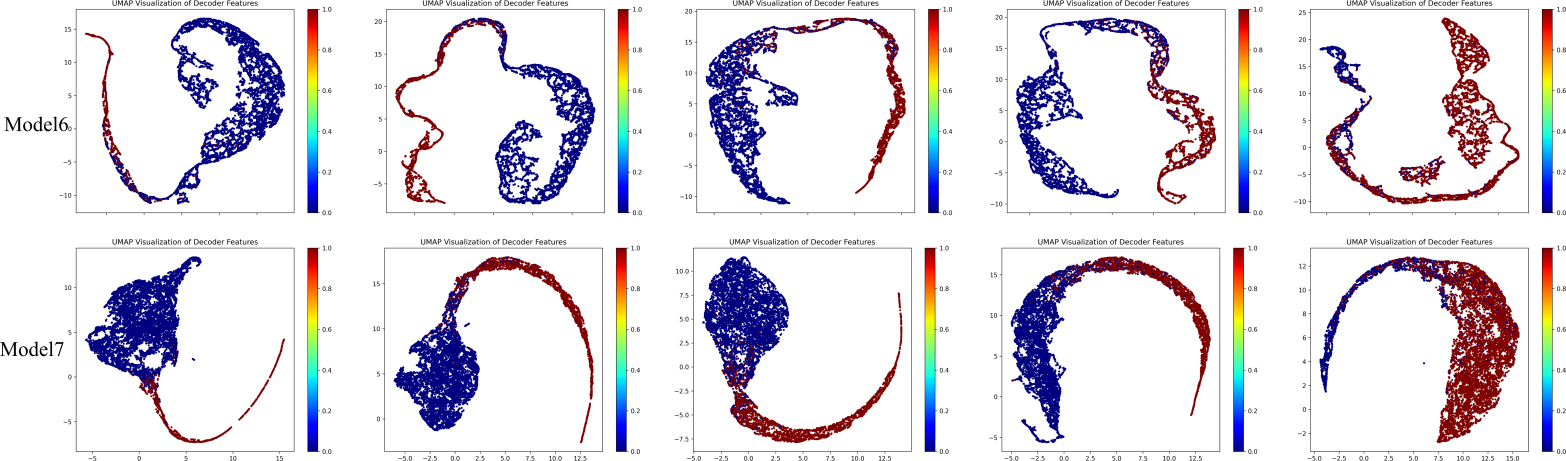
Feature visualization diagrams of model6 and model7.

Subsequently, we added the BAEM and MS-CL modules, leading to improvements in all five evaluation metrics, proving the effectiveness of these two methods. Notably, after incorporating the BAEM module, the HD95 decreased from 12.98 to 12.33, indicating its effectiveness in improving boundary segmentation accuracy. Finally, the best performance was achieved when all three modules were integrated into the basic model. To further illustrate the contribution of the proposed MS-CL module to class-specific feature discrimination, we visualized the feature representations obtained before the final output layer using UMAP, as shown in **Figure 9**. The visualizations correspond to Model 6 and Model 7. It can be observed that Model 7, which includes the MS-CL module, exhibits more compact intra-class clustering and clearer inter-class boundaries. These results suggest that MS-CL effectively enhances intra-class consistency and inter-class separability in the learned representations.

It is worth noting that the increase in the Jaccard index is more significant than that of the DSC, which can be attributed to the fact that the values reported in the table are the average results from five-fold cross-validation, rather than a single trial. From the formulas for DSC and Jaccard, we can derive the conversion formula between DSC and Jaccard for a single trial (Equation 15):


(15) $$DSC=\frac{2\cdot Jaccard}{1+Jaccard}$$

Thus, the relationship can be expressed as the following function: f(x)=x/(1+x) . If we have n points $x_{1},x_{2},\ldots ,x_{n}$ the function value at the mean of these points is: $f\left(\left(x_{1}+x_{2}+\ldots +x_{n}\right)/n\right)$ , and the mean of the function values can be expressed as: $\left(f(x_{1})+f(x_{2})+\ldots +f(x_{n})\right)/n$ . Thus, the difference Δ is defined as (Equation 16):


(16) $$\Delta =\frac{f(x_{1})+f(x_{2})+\ldots +f(x_{n})}{n}-f\left(\frac{x_{1}+x_{2}+\ldots +x_{n}}{n}\right)$$

The function f(x)=x/(1+x) is an increasing convex function, and therefore satisfies Jensen’s inequality (Equation 17):


(17) $$f\left(\frac{x_{1}+x_{2}+\ldots +x_{n}}{n}\right)\leq \frac{f(x_{1})+f(x_{2})+\ldots +f(x_{n})}{n}$$

Thus, the difference Δ is non-negative. Next, we perform a first and second-order Taylor expansion for each $x_{i}$ (Equation 18):


(18) $$f(x_{i})\approx f(\mu )+f^{\prime }(\mu )(x_{i}-\mu )+\frac{1}{2}f^{\prime \prime }(\mu ){\left(x_{i}-\mu \right)}^{2}$$

where $\mu =\left(\left(x_{1}+x_{2}+\ldots +x_{n}/n\right)\right)$ . Substituting these expansions into the difference calculation (Equation 19):


(19) $$\Delta =\frac{1}{n}\sum_{i=1}^{n}\left[f(\mu )+f^{\prime }(\mu )(x_{i}-\mu )+\frac{1}{2}f^{\prime \prime }(\mu ){\left(x_{i}-\mu \right)}^{2}\right]-f(\mu )$$

Since $\sum_{i=1}^{n}\left(x_{i}-\mu \right)=0$ , we obtain (Equation 20):


(20) $$\begin{array}{l} \Delta =\frac{1}{n}\sum_{i=1}^{n}\frac{1}{2}f^{\prime \prime }(\mu ){\left(x_{i}-\mu \right)}^{2}\\ \;=\frac{1}{2}f^{\prime \prime }(\mu )\cdot \frac{1}{n}\sum_{i=1}^{n}{\left(x_{i}-\mu \right)}^{2} \end{array}$$

Recognizing that $\sum_{i=1}^{n}{\left(x_{i}-\mu \right)}^{2}$ is the sum of the squared deviations of the samples, which is related to the standard deviation σ as follows (Equation 21):


(21) $$\sigma^{2}=\frac{1}{n}\sum_{i=1}^{n}{\left(x_{i}-\mu \right)}^{2}$$

Thus, the difference Δ can be expressed as (Equation 22):


(22) $$\Delta =\frac{1}{2}f^{\prime \prime }(\mu )\cdot n\cdot \sigma^{2}$$

This implies that the difference Δ is directly proportional to the square of the standard deviation (i.e., variance), and also proportional to the sample size n. In the quantitative results presented in this paper, we report the average results from five-fold cross-validation. Therefore, the smaller the difference in DSC and Jaccard, the lower the variance of the method. Based on the above analysis, we conclude that the incorporation of the three methods proposed in this paper significantly improves the model’s generalization performance.

### 4.6 Hyperparameter Study

To explore the effect of different scales and scale weights on segmentation performance in MS-CL, we conducted experiments with D values ranging from 1 to 5. Considering that smaller scales have higher resolution, the scale weights $\beta^{i},i\in \left(1,5\right)$ , should be negatively correlated with ii. The quantitative results for different hyperparameter settings are summarized in **Table 6**. The performance was better when D=2,3 compared to single-scale models, indicating that multi-scale information can help the model capture more diverse features. However, when D > 3, the performance dropped below that of single-scale models, suggesting that the bottleneck layer and high-scale information were not fully leveraged in the contrastive learning setup.

**Table 6:** Quantitative results of hyperparameter experiments using 20% labeled data on the OCDC dataset.

D	β^1^	β^2^	β^3^	β^6^	β^5^	OA(%)↑	AA(%)↑	DSC(%)↑	Jaccard(%)↑	HD_95_ ↓
1	1					88.42	84.42	81.64	70.59	12.39
2	0.7	0.3				88.25	87.56	81.34	70.82	12.22
3	0.7	0.2	0.1			88.26	87.92	81.69	71.20	12.29
3	0.6	0.3	0.1			88.72	88.26	82.23	75.82	12.10
4	0.4	0.3	0.2	0.1		88.17	82.43	81.10	70.00	12.44
5	0.4	0.2	0.2	0.1	0.1	87.44	83.44	82.36	71.72	12.03

The results report the average from five-fold cross-validation experiments.

## 5 Conclusions

This paper proposes a semi-supervised segmentation method for OPI based on transformation uncertainty and multi-scale contrastive learning. The method leverages a small amount of labeled data and a large amount of unlabeled data to jointly train the model, addressing the limitation of label scarcity and improving segmentation performance for OPI. In our method, we design a TB-UE approach that evaluates the model’s confidence on predictions for data transformed using different methods. This approach effectively mitigates the impact of semantic discrepancies between teacher model predictions and ground truth labels. Furthermore, we introduce a MS-CL approach, which enhances intra-class similarity and inter-class separability, while reducing the similarity between the teacher and student models, fostering more diverse feature representations. Additionally, we propose a boundary-aware U-Net model to capture the boundary information of OPI and integrate it with spatial features to improve segmentation accuracy. Extensive experiments on the OCDC dataset demonstrate the superiority of our method over fully supervised and other semi-supervised methods, providing new insights for alleviating data scarcity in pathology image segmentation.

Although the focus of this work is on methodological innovation, it is worth noting the potential clinical implications of the proposed model. Accurate and automated segmentation of pathological structures can provide critical support for pathologists by highlighting tumor boundaries and reducing diagnostic subjectivity. Furthermore, the proposed method is compatible with visual explanation tools such as class activation maps (CAMs), attention heatmaps, or uncertainty visualizations, which may enhance interpretability and foster trust in clinical practice. Integrating such models into digital pathology workflows could assist in pre-screening, prioritization, and quality assurance tasks. Future work may explore user studies or expert feedback to further validate the model’s utility in real-world diagnostic settings.

We also acknowledge several limitations of the current study. First, while our method achieves higher segmentation accuracy, it introduces additional computational cost due to the inclusion of the TB-UE and MS-CL modules. This results in increased model parameters and inference time. Future work will explore lightweight architectures or model compression strategies to reduce computational overhead while maintaining performance. Second, although the proposed approach is designed for oral squamous cell carcinoma, we have not yet verified its generalizability to other cancer sites. Evaluating the model’s transferability to other histopathological datasets—such as those related to lung, breast, or prostate cancer—will be a key direction in our future research. Lastly, while this paper emphasizes pixel-level annotation efficiency through semi-supervised learning, we recognize that obtaining fine-grained pathology annotations remains labor-intensive. To further reduce annotation costs, we plan to investigate weaker forms of supervision, such as image-level labels, scribbles, or pathologist sketches, potentially combined with active learning techniques.

## Data Availability

Publicly available datasets were analyzed in this study. This data can be found here: https://data.mendeley.com/datasets/9bsc36jyrt/1.
